# Isoliquiritigenin Inhibits Cigarette Smoke-Induced COPD by Attenuating Inflammation and Oxidative Stress via the Regulation of the Nrf2 and NF-κB Signaling Pathways

**DOI:** 10.3389/fphar.2018.01001

**Published:** 2018-09-20

**Authors:** Duo Yu, Xueshibojie Liu, Guangxin Zhang, Zhihui Ming, Tiejun Wang

**Affiliations:** ^1^Department of Radiotherapy, The Second Affiliated Hospital of Jilin University, Changchun, China; ^2^Department of Head and Neck Surgery, The Second Affiliated Hospital of Jilin University, Changchun, China; ^3^Department of Thoracic Surgery, The Second Affiliated Hospital of Jilin University, Changchun, China; ^4^Department of Stomatology, The Second Affiliated Hospital of Jilin University, Changchun, China

**Keywords:** cigarette smoke, lung inflammation, Nrf2, NF-κB, cytokines

## Abstract

Chronic obstructive pulmonary disease (COPD) is the major leading cause of disease with high-mortality worldwide. Cigarette smoke (CS) is a major factor for COPD. CS causes chronic inflammation and oxidative stress, which contributes to lung dysfunction in COPD. Isoliquiritigenin (ILG), a natural flavonoid derived from the root of liquorice, has been reported to possess antiinflammatory and antioxidant activity. In the present study, we tested the mechanism and protective effects of ILG on CS-induced COPD. Mice were exposed to CS for 2 h twice a day for 4 weeks. ILG was given orally 1 h before CS exposure twice a day for 4 weeks. The bronchial alveolar lavage fluid was collected to test the levels of inflammatory cytokines and the number of inflammatory cells. The lung tissues were obtained to evaluate the pathological changes, lung edema, myeloperoxidase (MPO) activity, malondialdehyde (MDA) level, as well as the expression of the nuclear factor-erythroid 2 (Nrf2) and nuclear factor κB (NF-κB) signaling pathways. The results showed that ILG reduced the infiltration of inflammatory cells and the production of inflammatory cytokines. ILG also reversed CS-induced lung pathological injuries, wet/dry ratio, MPO activity, and MDA level. Further research also showed that ILG dose-dependently up-regulated the expression of Nrf2 and down-regulated the expression of NF-κB signaling pathways induced by CS. In conclusion, ILG protected against CS-induced COPD by inhibiting inflammatory and oxidative stress via the regulation of the Nrf2 and NF-κB signaling pathways.

## Introduction

Chronic obstructive pulmonary disease (COPD) is a highly prevalent disease that is characterized by persistent respiratory symptoms due to small airway wall fibrosis, thickening, hypersecretion of mucus, oxidative stress, and inflammation in the lung (Zheng et al., 2008; Eapen et al., 2017; Vogelmeier et al., 2017a). Evidence indicates that COPD is the fourth leading cause of death in the world and is projected to become the third leading cause of death by 2020 (Lykkegaard et al., 2013; Li et al., 2017). Cigarette smoke (CS) is the most important risk factor for COPD (Du et al., 2017). Exposure to CS leads to the pathogenesis of COPD through increasing oxidative stress and inflammation in the lung (Chen et al., 2010; Du et al., 2017). Hence, it is necessary to develop new drugs with antioxidant and anti-inflammatory properties that also efficiently show down the progression of COPD.

Isoliquiritigenin (ILG), a natural flavonoid derived from the root of liquorice, has been widely used as the traditional Chinese medicine in western counties (Wu et al., 2016). ILG has various benefits, including antioxidant, anticancer, anti-inflammation, and anti-apoptotic properties (Traboulsi et al., 2015; Gao et al., 2017; Hou et al., 2017). Studies have demonstrated that ILG inhibits early brain impairments by regulating nuclear factor κB (NF-κB) and NLRP3 inflammasome pathways via triggering nuclear factor-erythroid 2 (Nrf2) activity (Zeng et al., 2017). Additionally, this inhibition of the NF-κB and NLRP3 inflammasome is an anti-inflammatory mechanism common to other flavonoids (Li et al., 2016; Menghini et al., 2016). ILG reduced the protein levels of Bax, cleaved-caspase-3, cleaved-caspase-9, and increased the level of anti-apoptotic Bcl-2 induced by IL-1β. ILG also inhibited NF-κB activation induced by IL-1β in chondrocyte-like ATDC5 cells (Ji et al., 2017). ILG has previously been shown to inhibit adipose tissue inflammation by inhibiting both NLRP3 inflammasome-dependent and independent mechanisms and reducing the adipose tissue fibrosis by regulating sensors of innate immunity (Watanabe et al., 2016). However, it remains unclear whether ILG is effective for treating COPD. Thus, the mechanism and protective effect of ILG on CS-induced COPD were tested in the present study.

## Materials and Methods

### Animals

Sixty C57BL/6N male mice, 6–8 weeks, were purchased from Experimental Animal Center of Baiqiuen Medical College of Jilin University. The mice were housed under pathogen-free conditions with sufficient water and food and subjected to a light-dark cycle of 12 h. All experimental procedures complied with the requirements of the Animal Ethics Committee of Jilin University (Permit Number: 20170612). All procedures were approved by the Guide for the Care and the Use of Laboratory Animals manual published by the US National Institutes of Health.

### Materials

Isoliquiritigenin was purchased from Sigma (Signma-Aldrich, United States). Cigarettes were purchased from Tobacco Group Company Limited. Myeloperoxidase (MPO) and malondialdehyde (MDA) assay kits were purchased from Jiancheng Bioengineering Institute (Nanjing, China). TNF-α and IL-1β ELISA kits were purchased from eBioscience (San Diego, CA, United States). Antibodies against NF-κB p65, p-NF-κB p65, IκBα, p-IκBα, Nrf2, and β-actin were purchased from Cell Signaling Technology Inc., (Beverly, MA, United States). All other chemicals were of reagent grade.

### Animal Model Administration and Treatment

Sixty mice were randomly divided into five groups and each group contained 12 mice, including the control group, the CS group, the ILG (10, 20, and 30 mg/kg) + CS groups. The CS-induced COPD model was established as described previously (Luo et al., 2017). Mice were exposed to CS for 2 h twice a day for 4 weeks. The control group was exposed to room air. Mice from the ILG + CS groups were given 10, 20, and 30 mg/kg ILG (diluted in PBS) orally 1 h before CS administration twice a day for 4 weeks. The doses of ILG used in this study were based on a previous study (Liu et al., 2017). Mice from the control group were treated with the same volume of sterile PBS. Mice were sacrificed 4 weeks later. Bronchial alveolar lavage fluid (BALF) and lung tissues were collected tor testing.

### BALF Collection

The BALF was collected as previously described (Xueshibojie et al., 2016). After the last exposure of CS, mice were exsanguinated, and the thorax was opened. Five hundred microliter of PBS was injected into, retrieved from the trachea, and repeated three times. The samples were centrifuged at 3000 rpm for 10 min, and the supernatant was stored at -80°C until use.

### Lung Pathological Evaluation

The lung tissues that were not subjected to BALF were used to evaluate pathological changes. The samples were fixed in 10% formalin for 48 h and dehydrated in a series of graded ethanol, embedded in paraffin, cut into 4 μm sections. The section was stained with haematoxylin and eosin for pathological assay.

### Lung Wet/Dry Ratio Assay

The lung wet/dry ratio was used as an index of lung edema. The upper lobe of the right lung was immediately weighed after collection and recorded as wet weight. The lung tissues were then placed into an oven at 60°C for 72 h and weighed as dry weight. The lung wet/dry ratio was calculated by dividing the wet by the dry weights.

### MPO and MDA Assay

Mice were sacrificed, and the lung tissues were collected. The samples were homogenized and dissolved in extraction buffer. To test the number of neutrophils and the level of lipid peroxidation in the lung tissues, MPO and MDA levels were tested by MPO and MDA detection kits in accordance with the manufacturer’s protocols.

### Inflammatory Cytokines Assay in BALF

The levels of inflammatory cytokines TNF-α and IL-1β in BALF were tested by TNF-α and IL-1β ELISA kits according to the manufacturer’s instruction (Xueshibojie et al., 2016). The standard curve was established according to the standards provided by the kits. The sensitivity for TNF-α and IL-1β ELISA kits were 31.3 and 25 pg/mL, respectively. Inter-assay and Intra-assay coefficients of variation were 4.1–8.3% and 2.7–8.3%, respectively.

### Inflammatory Cell Count Assay in BALF

The numbers of total cells, neutrophils, and macrophages in the BALF were tested in this study as previously described (Xueshibojie et al., 2016). Briefly, BALF was collected and centrifuged at 3000 rpm at 4°C for 10 min. The cell pellets were resuspended in 0.2 mL PBS, and the total cell counts were performed using a hemocytometer. For differential cells counts, cytospin slides were prepared, stained with Wright’s stain, and 300 cells were counted per slide.

### Western Blot Assay

The total protein in the lung tissues were analyzed by Western blot as previously described (Cheng et al., 2015). The protein concentration was tested by BCA kit. The protein was separated in 12% Tris-glycine SDS polyacrylamide gel and transferred onto polyvinylidene fluoride membranes. After incubation for 2 h with 3% BSA at room temperature, the membranes were incubated with anti-NF-κB p65, anti-NF-κB p-p65, anti-IκBα, anti-pIκB, anti-Nrf2, and anti-β-actin antibodies at 4°C overnight. After washing three times, the membranes were incubated with secondary antibody at room temperature for 45 min. The membranes were washed three times again, and the membranes were developed with an enhanced chemiluminescence Western blotting detection system.

### Statistical Analysis

Data were analyzed using the SPSS statistical software, version 17.0. Values were expressed as the mean ± standard deviation (SD) of three independent experiments. One-way analysis of variance (ANOVA) followed by Tukey’s *post hoc* method was used for multiple comparisons among groups. A *p-*value of less than 0.05 was considered statistically significant.

## Results

### ILG Prevents CS-Induced Lung Pathologic Changes

**FIGURE 1:**
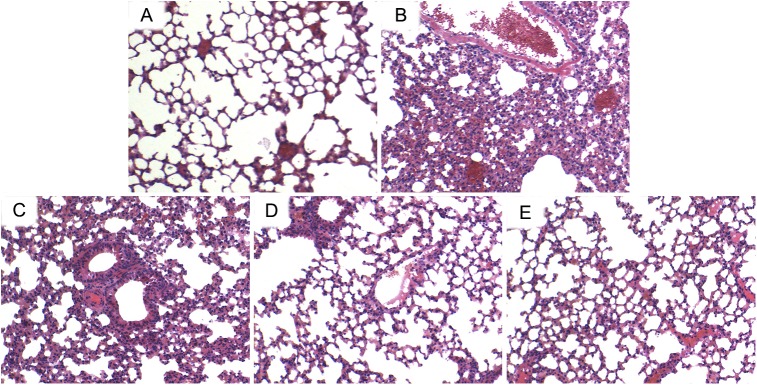
Effects of isoliquiritigenin (ILG) on CS-induced histological changes in lung tissues. Representative histological changes of lung obtained from mice of different groups. **(A)** Control group, **(B)** CS group, **(C)** CS+ ILG (10 mg/kg) group, **(D)** CS + ILG (20 mg/kg) group, **(E)** CS + ILG (30 mg/kg) group (hematoxylin and eosin staining, magnification 200×).

H & E staining was used to assess the pathological changes of the lung tissues. The results indicated that the lung tissues from CS group showed markedly pathological changes, including a large number of infiltrating inflammatory cells, and intra-alveolar edema compared with lung tissues from the control group. However, administration of ILG significantly reduced these changes induced by CS (**Figure 1**).

### ILG Attenuates CS-Induced Lung Wet/Dry Ratio

**FIGURE 2:**
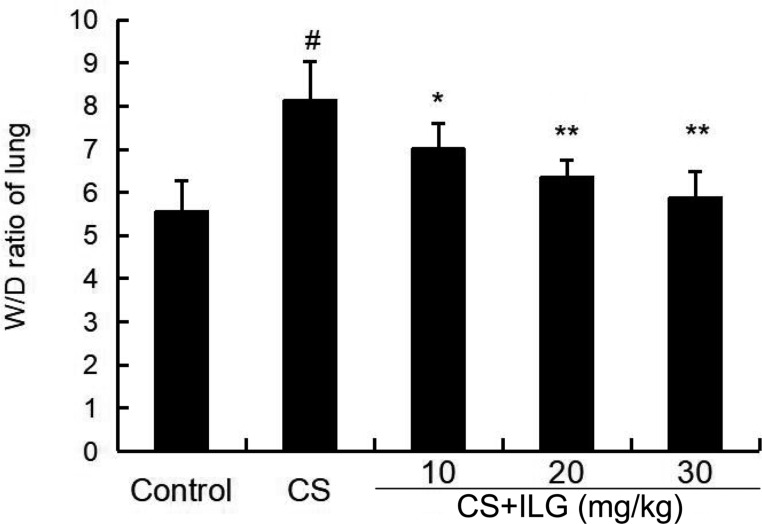
Effects of isoliquiritigenin on lung edema of cigarette smoke (CS)-induced lung inflammation in mice. The values presented are the means ± SEM of three independent experiments. #*p* < 0.01 vs. control group, ^∗^*p* < 0.05, ^∗∗^*p* < 0.01 vs. CS group.

To evaluate the severity of lung edema, the lung wet/dry ratio was tested in the present study. The results showed that lung wet/dry ratio was obviously increased after CS exposure. Furthermore, the lung wet/dry ratio was attenuated by ILG in a dose-dependent manner (**Figure 2**).

### ILG Attenuates CS-Induced MPO Activity in the Lung

**FIGURE 3:**
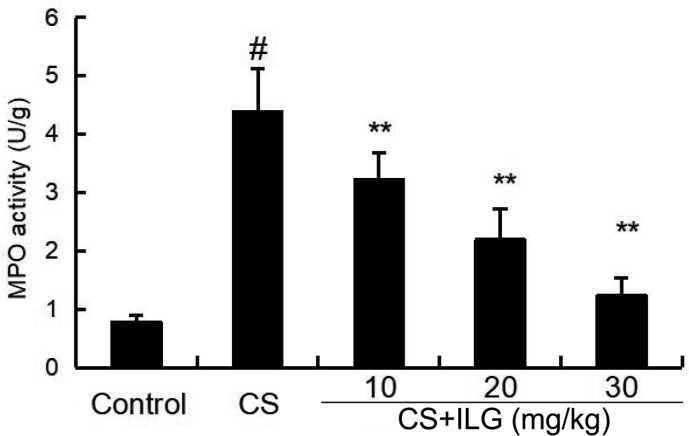
Effects of isoliquiritigenin on myeloperoxidase activity of cigarette smoke (CS)-induced lung inflammation in mice. Lung tissues were collected at 24 h after the last CS exposure. The values presented are the means ± SEM of three independent experiments. #*p* < 0.01 vs. control group, ^∗∗^*p* < 0.01 vs. CS group.

MPO activity was used to test the accumulation of neutrophils in the lung tissues. As shown in **Figure 3**, MPO activity was significantly increased after CS exposure. And MPO activity markedly reduced after ILG treatment in a dose-dependent manner.

### ILG Attenuates CS-Induced MDA Level in the Lung

**FIGURE 4:**
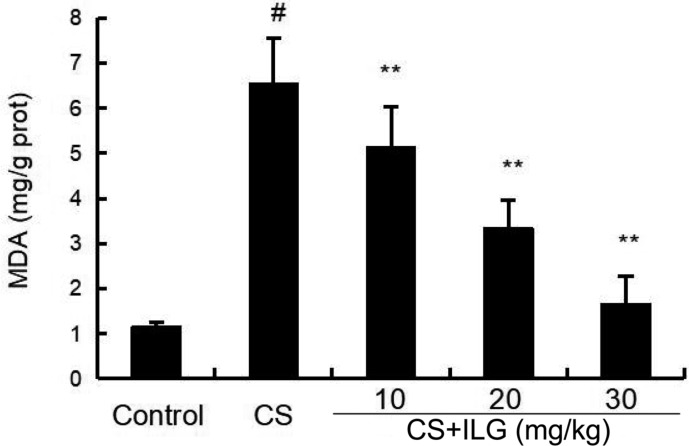
Effects of isoliquiritigenin on malondialdehyde production of cigarette smoke (CS)-induced lung inflammation in mice. Bronchial alveolar lavage fluid was collected at 24 h after the last CS exposure. The values presented are the means ± SEM of three independent experiments. #*p* < 0.01 vs. control group, ^∗∗^*p* < 0.01 vs. CS group.

As shown in **Figure 4**, increased levels of MDA were found in the lung tissues from the CS group compared with the lung tissues from the control group. Compared to the CS group, ILG co-treatment reduced CS-induced lipid peroxidation as indicated by inhibiting the level of MDA in the lung tissues.

### ILG Attenuates CS-Induced Inflammatory Cytokines Levels in BALF

**FIGURE 5:**
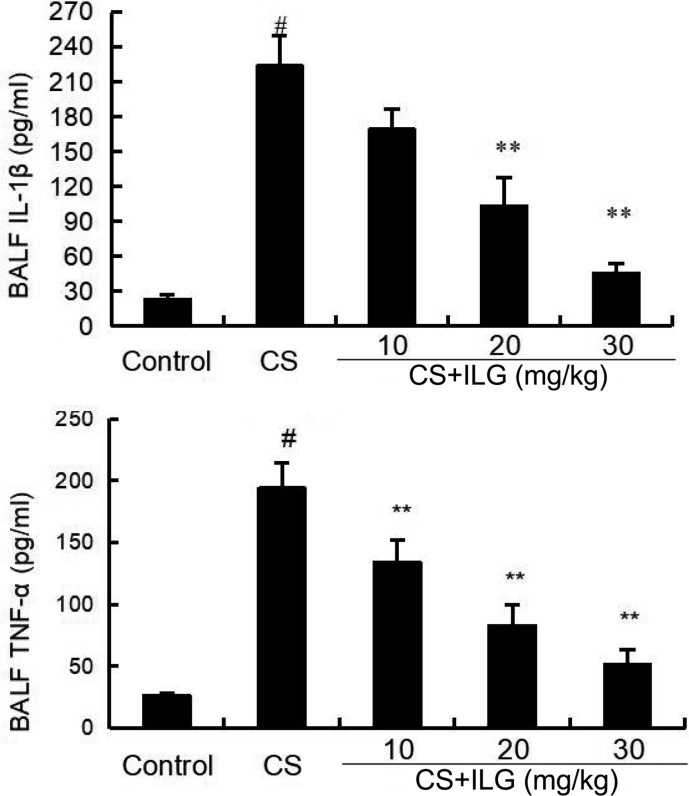
Effects of isoliquiritigenin on TNF-α and IL-1ß production in the bronchial alveolar lavage fluid of cigarette smoke (CS)-induced lung inflammation in mice. The production of TNF-α and IL-1ß were detected by ELISA. The values presented are the means ± SEM of three independent experiments. #*p* < 0.01 vs. control group, ^∗∗^*p* < 0.01 vs. CS group.

The effects of ILG after CS exposure on the levels of inflammatory cytokines TNF-α and IL-1β levels were analyzed by ELISA. The results showed that the levels of TNF-α and IL-1β in the BALF were significantly increased in the CS group compared with those in the control group. However, treatment with ILG reversed the levels of TNF-α and IL-1β induced by CS (**Figure 5**).

### ILG Attenuates CS-Induced Inflammatory Cell Counts in the BALF

**FIGURE 6:**
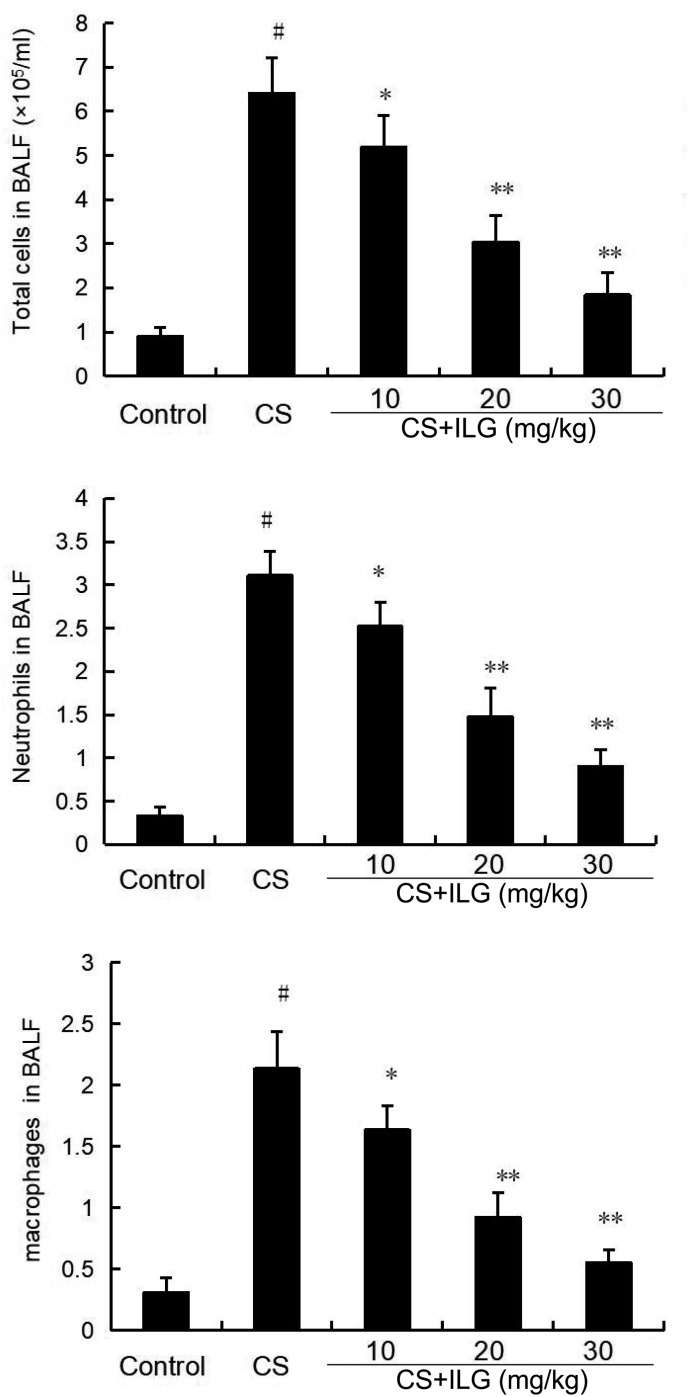
Effects of isoliquiritigenin on inflammatory cells count in the bronchial alveolar lavage fluid of cigarette smoke (CS)-induced lung inflammation in mice. The values presented are the means ± SEM of three independent experiments. #*p* < 0.01 vs. control group, ^∗^*p* < 0.05 and ^∗∗^*p* < 0.01 vs. CS group.

The total number of inflammatory cells, neutrophils, and macrophages in the BALF were calculated after CS exposure. The results showed that CS exposure markedly increased the number of total cells, neutrophils, and macrophages compared the control group. ILG treatment at the doses of 10, 20, and 30 mg/kg markedly reduced the number of total cells, neutrophils, and macrophages (**Figure 6**).

### ILG Attenuates CS-Induced NF-κB Activation in the Lung

**FIGURE 7:**
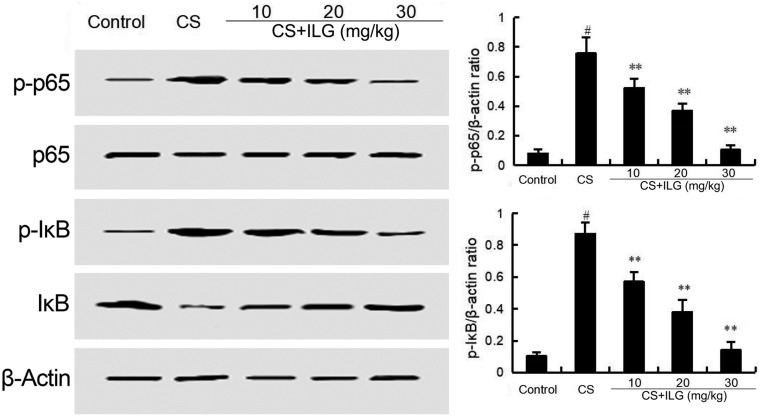
Isoliquiritigenin inhibits cigarette smoke (CS)-induced NF-κB activation in lung tissues. The values presented are the means ± SEM of three independent experiments. #*p* < 0.01 vs. control group, ^∗∗^*p* < 0.01 vs. CS group.

The effects of ILG on CS-induced NF-κB activation was tested by Western blot analysis. The results showed that the phosphorylation of NF-κB p65 and IκBα were markedly increased in the CS group compared with the control group. Treatment of ILG dose-dependently reduced the expression of NF-κB p-p65 and p-IκBα induced by CS (**Figure 7**).

### ILG Upregulates CS-Induced Nrf2 Activation in the Lung

**FIGURE 8:**
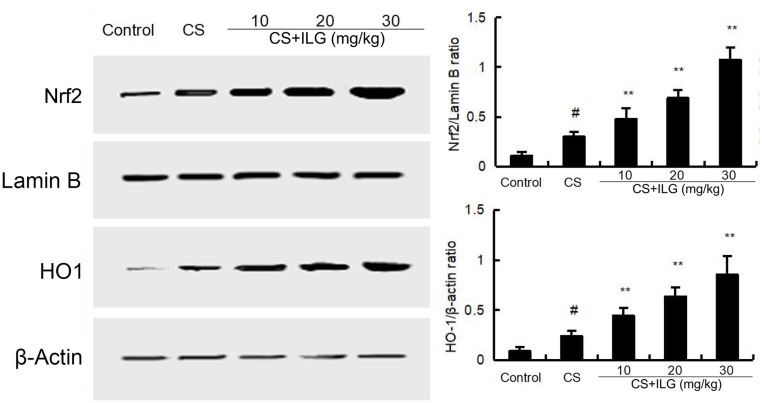
Effects of isoliquiritigenin on Nrf2 and HO-1 expression in lung tissues. The values presented are the means ± SEM of three independent experiments. #*p* < 0.01 vs. control group, ^∗∗^*p* < 0.01 vs. cigarette smoke group.

To study the anti-oxidant effects of ILG, the protein expression of Nrf2 and HO-1 were tested by Western blot. As shown in **Figure 8**, the levels of Nrf2 and HO-1 were up-regulated after CS exposure. This up-regulation of Nfr2 and HO-1 were further increased by ILG in a dose-dependent manner.

## Discussion

Chronic obstructive pulmonary disease is a life-threatening syndrome associated with high morbidity and mortality rates around the world (Cheng et al., 2015; Vogelmeier et al., 2017b). The major characteristics of COPD are chronic inflammation in the lung that results in reduced airflow. CS is thought to be a key factor that contributes to COPD. CS is an important inducer of oxidative stress and the inflammatory response, which leads to the pathogenesis of COPD (van der Vaart et al., 2004; Churg et al., 2008; Cheng et al., 2015). ILG, extracted from the root of liquorice, has been widely used to cure many inflammatory diseases, because of its anticancer, antioxidant, and anti-inflammatory properties (Zeng et al., 2017; Chen et al., 2018; Li et al., 2018; Na et al., 2018). In the present study, we tested the mechanism and protective effects of ILG on CS-induced COPD. The results showed that in the lung, ILG dose-dependently inhibited pathological damage, wet/dry ratio, MPO activity, MDA level, production of inflammatory cytokines, and number of inflammatory cells in BALF induced by CS. To test the mechanism of ILG on CS-induced COPD, activation of the Nrf2 and NF-κB signaling pathways were tested. The results showed that treatment of ILG significantly inhibited the activation of NF-κB and promoted the activation of Nrf2 induced by CS. These data demonstrated that ILG protected against CS-induced COPD by inhibiting inflammation and oxidative stress via the regulation of Nrf2 and NF-κB signaling pathway activation.

Inflammatory cytokines TNF-α and IL-1β play an important role in COPD. Over-production of TNF-α and IL-1β could amplify the inflammatory response and lead to lung dysfunction (Churg et al., 2002; Cheng et al., 2015). In addition, these cytokines can induce the recruitment of neutrophils to the lung, causing chronic bronchial inflammation and emphysema (Churg et al., 2002; Cheng et al., 2015). Our results showed that ILG significantly inhibited the levels of inflammatory cytokines TNF-α and IL-1β induced by CS. Increased MPO activity is a major indicator of neutrophils (Shen et al., 2014; Shin et al., 2016). In the present study, increased MPO activity was found in the CS-treated groups. And treatment with ILG markedly reduced MPO activity induced by CS. To test the effects of ILG on lung edema, the lung wet/dry ratio was measured in the present study. ILG dose-dependently reduced lung wet/dry ratio induced by CS. In addition, inflammatory cells, including neutrophils, and macrophages play an important role in the inflammatory response (Moffet et al., 2016; Xu et al., 2016). In the present study, we found that total number of cells, neutrophils, and macrophages were significantly increased in the BALF from the CS groups compared with the control group. Administration of ILG reduced the total number of cells, neutrophils, and macrophages induced by CS in a dose-dependent manner. These data proved that ILG has an ability to protect lungs from CS-induced COPD.

Neutrophils and macrophages are the major inflammatory cells that generate ROS and inflammatory mediators that accelerate injury in the lung (Forman and Torres, 2002). Oxidative stress also plays a critical role in the development of COPD (Zuo et al., 2014). Increased lipid peroxidation was observed in mice of CS-induced COPD, and the increased lipid peroxidation could also induce lung injury (Thayaparan et al., 2016). In this study, we assessed the lipid peroxidation by measuring MDA content. Our results showed that ILG significantly inhibited CS-induced MDA production. Nrf2 is an important transcription factor that is involved in regulating the antioxidant response. Evidence suggests that the expression of Nrf2 play an important role in CS-induced COPD (Rangasamy et al., 2004; Singh et al., 2009). Up-regulation of Nrf2 improved CS-induced oxidant stress in rat lungs. In addition, studies have reported that NF-κB activation is associated with the development of COPD. Activation of Nrf2 protect against CS-induced lung inflammation by regulating NF-κB activation (Cho et al., 2002; Gebel et al., 2010). To study the protective mechanism of ILG on CS-induced COPD, the expression of NF-κB and Nrf2 were tested in the present study. The data showed that CS exposure activated NF-κB and Nrf2 signaling pathways in the lung. And these changes were inhibited by ILG treatment. The above results suggested that ILG inhibited CS-induced COPD through inhibiting inflammatory reaction and oxidative stress, which are involved in the regulation of the Nrf2 and NF-κB signaling pathway.

## Conclusion

In conclusion, our results indicated that ILG significantly inhibited CS-induced COPD, and that the mechanism of this effect might involve regulation of the Nfr2 and NF-κB signaling pathways.

## Author Contributions

DY, ZM, and TW designed the experiments. DY, XL, GZ, and ZM did the experiments. TW analyzed the data. DY and XL wrote the article.

## Conflict of Interest Statement

The authors declare that the research was conducted in the absence of any commercial or financial relationships that could be construed as a potential conflict of interest.
